# 

**DOI:** 10.1192/bjb.2022.78

**Published:** 2023-10

**Authors:** Sarah Mehmood, Annabel Price

**Affiliations:** Medical student, School of Medicine, Faculty of Medicine and Health, University of Leeds, UK; Consultant Liaison Psychiatrist, Department of Psychological Medicine, Cambridgeshire and Peterborough Mental Health Partnership NHS Trust, Fulbourn, UK. Email: annabel.price@cpft.nhs.uk

**Figure:**
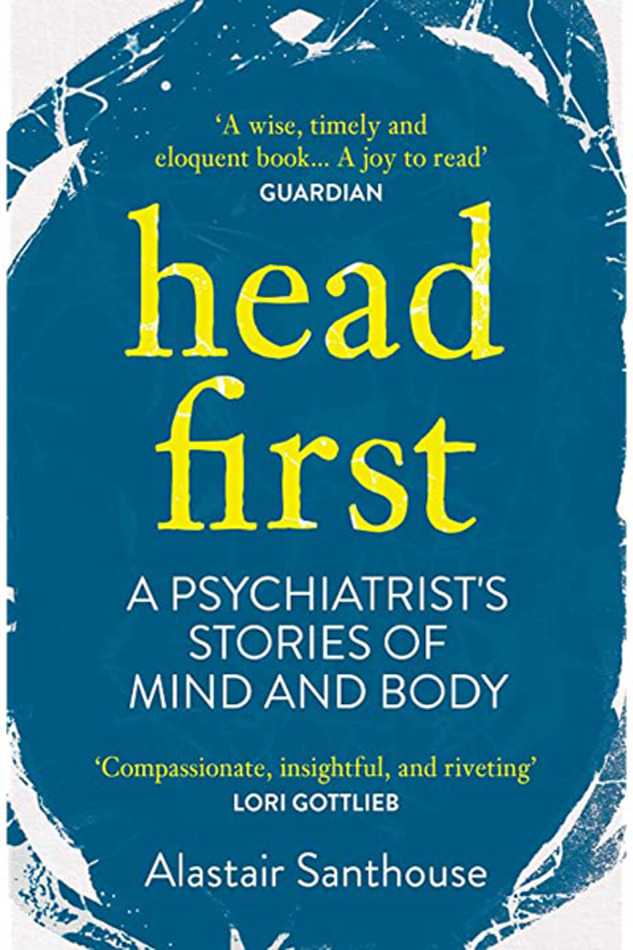

Painting vivid, exquisitely detailed pictures, this book delivers poignant lessons through human stories and personal memoir told with self-deprecating humour and humility. An emphatic, thoughtful work, *Head First* takes the reader to hidden crevices and open canyons – challenging our thoughts on medicine and exploring the far-reaching consequences of separating ‘mental’ from ‘physical’. In ‘Stigma’ for example, we meet sisters Pearl and Sadie, whose isolation shows us the deep consequences of self-stigmatisation. Simon, who we meet in ‘Melancholia’, experiences the fraught loneliness of depression, and Gary's frequent and baffling presentations to hospital in ‘Medical mysteries’ teach us that the answer is not always revealed by ever more medical tests. The patient stories are contextualised with wide-ranging and erudite discussion on subjects as diverse as autonomy, personality and the meaning of an evidence base in medicine.

Dr Santhouse reflects on his years at medical school and his utter bewilderment at the question ‘is medicine an art or a science?’, believing then that it could be nothing but a science. His striking memoir, however, is a powerful argument for medicine as both. To look beyond the obvious, to understand each patient in their own messy realities, in tears, pain and joy, is a privilege of medicine that the author so perfectly enunciates.

This book recaptures an often neglected understanding of the person as a whole, while being rooted firmly in a contemporary understanding of integrated psychological medicine. It is accessible to the non-clinical (or clinical novice) reader, but for more experienced or even expert readers it offers a richer understanding of the mind–body interface and an invitation to step back and take a fresh look at medicine.

